# Machine learning predicts peak oxygen uptake and peak power output for customizing cardiopulmonary exercise testing using non-exercise features

**DOI:** 10.1007/s00421-024-05543-x

**Published:** 2024-07-03

**Authors:** Charlotte Wenzel, Thomas Liebig, Adrian Swoboda, Rika Smolareck, Marit L. Schlagheck, David Walzik, Andreas Groll, Richie P. Goulding, Philipp Zimmer

**Affiliations:** 1 Institute for Sport and Sport Science, Performance and Health (Sports Medicine), TU Dortmund University, Dortmund, Germany; 2 Institute for Computer Science, Department of Artificial Intelligence, TU Dortmund University, Dortmund, Germany; 3 Institute for Training Optimization for Sport and Health, iQ Athletik, Frankfurt am Main, Germany; 4 Department of Statistics, Statistical Methods for Big Data, TU Dortmund University, Dortmund, Germany; 5 Faculty of Behavioral and Movement Sciences, Department of Human Movement Sciences, Vrije Universiteit Amsterdam, Amsterdam Movement Sciences, Amsterdam, The Netherlands

**Keywords:** Cardiopulmonary exercise testing, Machine learning, Peak oxygen uptake, Peak power output, Prediction

## Abstract

**Purpose:**

Cardiopulmonary exercise testing (CPET) is considered the gold standard for assessing cardiorespiratory fitness. To ensure consistent performance of each test, it is necessary to adapt the power increase of the test protocol to the physical characteristics of each individual. This study aimed to use machine learning models to determine individualized ramp protocols based on non-exercise features. We hypothesized that machine learning models will predict peak oxygen uptake ($\dot{V}$O_2peak_) and peak power output (PPO) more accurately than conventional multiple linear regression (MLR).

**Methods:**

The cross-sectional study was conducted with 274 (♀168, ♂106) participants who performed CPET on a cycle ergometer. Machine learning models and multiple linear regression were used to predict $\dot{V}$O_2peak_ and PPO using non-exercise features. The accuracy of the models was compared using criteria such as root mean square error (RMSE). Shapley additive explanation (SHAP) was applied to determine the feature importance.

**Results:**

The most accurate machine learning model was the random forest (RMSE: 6.52 ml/kg/min [95% CI 5.21–8.17]) for $\dot{V}$O_2peak_ prediction and the gradient boosting regression (RMSE: 43watts [95% CI 35–52]) for PPO prediction. Compared to the MLR, the machine learning models reduced the RMSE by up to 28% and 22% for prediction of $\dot{V}$O_2peak_ and PPO, respectively. Furthermore, SHAP ranked body composition data such as skeletal muscle mass and extracellular water as the most impactful features.

**Conclusion:**

Machine learning models predict $\dot{V}$O_2peak_ and PPO more accurately than MLR and can be used to individualize CPET protocols. Features that provide information about the participant's body composition contribute most to the improvement of these predictions.

**Trial registration number:**

DRKS00031401 (6 March 2023, retrospectively registered).

**Supplementary Information:**

The online version contains supplementary material available at 10.1007/s00421-024-05543-x.

## Introduction

Cardiopulmonary exercise testing (CPET) on a cycle ergometer is widely applied in endurance sports as well as in clinical settings. It provides a comprehensive insight into integrated cardiopulmonary function within a single laboratory session. In particular, maximal oxygen uptake ($\dot{V}$O_2max_) reflects the integrated capacity of the cardiopulmonary and neuromuscular systems to take up, transport, and utilize oxygen during exercise (Poole and Jones 2017). It thus represents the greatest attainable rate of aerobic adenosine triphosphate generation and is a marker of exercise capacity (Bassett and Howley 2000). In addition to $\dot{V}$O_2max_, peak power output (PPO) is typically used as a measure of exercise capacity. It is quantified in external units of power output, thus requiring less specialist equipment. Both outcomes are strongly predictive of all-cause mortality and the risk of developing chronic diseases (Ross et al. 2016). They can be applied to manage exercise training by determining training intensity and to validate and monitor the success of training interventions (Myers 2005).

To measure valid and interpretable CPET values on a cycle ergometer, the appropriate rate of increase in power and associated test duration are relevant factors. A too-rapid increase and a short test duration may lead to hyperventilation, lack of determinability of the gas exchange threshold (Glaab and Taube 2022), and premature end of the test due to the occurrence of task failure prior to the attainment of $\dot{V}$O_2max_ (Hill et al. 2002)_._ Too slow increase in power and a long test duration could result in insufficient $\dot{V}$O_2_ drive to reach $\dot{V}$O_2max_. This may lead to test termination due to factors not typically associated with reaching the tolerance limit during severe exercise (e.g. peripheral muscle fatigue, accumulation of metabolic products associated with fatigue, etc.) (Vanhatalo et al. 2010; Burnley et al. 2012). Consequently, an inappropriate increase in power can lead to early or delayed termination of CPET and failure to accurately determine $\dot{V}$O_2max_. Due to the challenge of attaining $\dot{V}$O_2max_, peak oxygen uptake ($\dot{V}$O_2peak_) is alternatively employed as an indicator of physical performance, representing the highest $\dot{V}$O_2_ value determined during the CPET.

To reach peak performance values, the test protocol should be precisely adjusted to the participant achieving voluntary exhaustion within the recommended 8 to 12 min (Buchfuhrer et al. 1983; American College of Sports Medicine 2021). Standardization of test duration across participants would ensure the comparability of results between different test facilities or clinical environments and lead to optimized processes. To enable valid completion of each test, it is necessary to adapt the power increase to the physical characteristics of every individual. It would be advantageous if these characteristics are non-exercise features that are convenient to collect in daily practice before conducting a CPET and are expected to have an impact on $\dot{V}$O_2peak_ and PPO. Furthermore, appropriate prediction models are important for customizing the protocol. In addition to $\dot{V}$O_2peak_, which is commonly used as an outcome parameter (Myers et al. 2001; da Silva et al. 2012; Cunha et al. 2015), PPO depicts a further outcome parameter that can be collected with less equipment.

Previous work investigating protocol adaptations for CPET included small (Saengsuwan et al. 2017) or homogeneous (Myers et al. 1994; Cunha et al. 2015) populations and usually used conventional linear predictive models (Myers et al. 1994, 2001; da Silva et al. 2012; Saengsuwan et al. 2017). This may lead to a possible overestimation of the explanatory power of the models. Indeed, the American College of Sports Medicine recommends formulas for predicting $\dot{V}$O_2max_ based on multiple linear regression (American College of Sports Medicine 2021). Thus, potential non-linear relationships between the features and the outcome parameter cannot be identified. Meanwhile, several machine learning models, like decision tree (Song and Lu 2015), random forest (Breiman 2001), *k*-nearest-neighbor (Sreevalsan-Nair 2020), and gradient boosting regression (Friedman 2001), are able to capture non-linear patterns. They can incorporate many features, and deal with heterogeneous data and the associated outliers (Friedman 2001; Singh et al. 2016). Due to this background, this work aims (1) to compare two different non-exercise feature sets to predict $\dot{V}$O_2peak_ and PPO using four machine learning models and one linear model and (2) to identify the most impactful features to adapt the power increase to the physical conditions of each participant on a cycle ergometer. We hypothesize that machine learning models provide more accurate predictions of $\dot{V}$O_2peak_ and PPO than the conventional multiple linear regression technique.

## Materials and methods

### Participants

The cross-sectional study included *n* = 274 (♀168, ♂106) participants who were at least 18 years old and physically able to perform CPET. Participants were asked not to engage in vigorous exercise or drink alcohol or coffee for 24 h and not to eat for 2 h prior to the measurement. Participants were excluded from the study if they had a pacemaker, an acute infection, or an orthopedic injury. Female subjects were also excluded if they were pregnant.

### Procedures

Participants’ demographic and anthropometric data were collected. Subsequently, body composition was assessed by bioelectrical impedance analysis (BIA) (SECA mBCA 525), and handgrip strength measurement (Jamar hand dynamometer hydraulic) was conducted. Afterward, participants answered questionnaires on physical activity level (PAL) (Godin and Shephard 1985; Armstrong and Bull 2006) and sleep quality (Buysee et al. 1989). The CPET was performed in an upright position on a cycle ergometer (CORTEX Bike M). The test began with a one-minute resting measurement and a 2-min period of baseline cycling at 25 watts (W) for females and 50 W for males. Subsequently, we increased the workload by 15, 20 or 25 W/min for females and 20, 25 or 30 W/min for males, depending on PAL category and body-mass-index (BMI) (supplements Table A). The power increased continuously until participants were no longer able to maintain a frequency above 60 revolutions per minute. This was followed by a recovery phase of 3 min at 25 W for females or 50 W for males.

Respiratory gas exchange and ventilation were measured continuously on a breath-by-breath basis via spiroergometry (CORTEX METAMAX^®^ 3B). Heart rate (HR) was monitored permanently via a bluetooth chest strap (Polar H10). The highest 15-s average determined by the software (CORTEX MetaSoft^®^ Studio) during the CPET was regarded as peak $\dot{V}$O_2_ ($\dot{V}$O_2peak_), respiratory exchange ratio (RER) and maximal HR (HR_max_). The highest power output achieved during the CPET prior to exhaustion was considered the PPO. Time to exhaustion (TTE) was defined by the time of exercise test minus baseline and recovery periods. To ensure that most participants had achieved their full capacity, they had to reach a $\dot{V}$O_2_ plateau with 150 ml/min difference between the last two 30-s intervals or meet two of the following three criteria at the time of $\dot{V}$O_2peak_: (1) a RER ≥ 1.1, (2) a rating of perceived exertion > 17 on the 6–20 scale and (3) a HR_max_ within 10 beats/min of the age-predicted HR_max_ (Tanaka et al. 2001).

### Feature selection

The selection of non-exercise features for the prediction of $\dot{V}$O_2peak_ and PPO was based on possible associations with physical performance (Wier et al. 2006; Schembre and Riebe 2011; Booth et al. 2012; Antunes et al. 2017; Saengsuwan et al. 2017; Przednowek et al. 2018; Langer et al. 2020; American College of Sports Medicine 2021; Shen et al. 2022). The small feature set comprised 15 features that are convenient to assess in practice and have a low time requirement. These include anthropometric and demographic data, as well as self-perceived health status and activity level. The big feature set was extended by body composition variables, handgrip strength, and questionnaires (Godin and Shephard 1985; Buysee et al. 1989; Armstrong and Bull 2006), and comprised 41 features. All features are listed in Table 1 and explained in more detail in the supplements (Table B).

**Table 1:** Small and big feature set

Feature set	Features
Small feature set	Age (years), sex, smoking, smoking behavior (years), chronic diseases, allergies, supplements, medications, PAL, weight (kg), height (m), BMI, waist circumference (cm), hip circumference (cm), waist-hip-ratio
Big feature set	Age (years), sex, smoking, smoking behavior (years), chronic diseases, allergies, supplements, medications, PAL, weight (kg), height (m), BMI, waist circumference (cm), hip circumference (cm), waist-hip-ratio, FM (%), FM (kg), FFM (%), FFM (kg), SMM (%), SMM (kg), SMM torso (kg), SMM torso (%), SMM legs (kg), SMM legs (%), TBW (%), TBW (L), ECW (%), ECW (L), ECW/TBW (%), total energy expenditure (kcal/day), resting energy expenditure (kcal/day), phase angle (°), handgrip strength (kg), handgrip strength (%), MET, PAL category, Godin score, Godin category, PSQI score, PSQI category

*PAL* physical activity level, *BMI* body-mass-index, *FM* fat mass, *FFM* fat free mass, *SMM* skeletal muscle mass, *TBW* total body water, *ECW* extracellular water, *MET* metabolic equivalent task, *PSQI* Pittsburgh Sleep Quality Index

### Applied machine learning algorithms

Multiple linear regression (Jobson 1991) as a conventional model and four different supervised machine learning models were used to predict the $\dot{V}$O_2peak_ and PPO, each utilizing the two feature sets. Table 2 lists the multiple linear regression, and the machine learning models, their description, and the reasons for selection.

**Table 2:** Description of the supervised machine learning models and justification of the selection

Models	Description	Reason
Multiple linear regression	Multiple linear regression is used to model a continuous variable and make predictions. The independent features and the dependent feature must be linearly related to fit a straight line to the data set (Ray 2019).	Multiple linear regression is the most commonly used statistical technique especially in exercise physiology (Jobson 1991; Akay and Abut 2015). It provides insights into the relationship between independent and dependent features. Multiple linear regression is a simple and comprehensible method (Ray 2019).
Decision tree	In decision tree, actions are executed by if–then conditions by using a single attribute for splitting. The data are split in the nodes and the decisions are in the leaves (Breiman et al. 1984; Ray 2019).	Decision tree is easy to implement. It can handle categorical and quantitative values and is easy to interpret and visualize (Ray 2019).
Random forest	The random forest contains several decision trees that are applied in parallel to different subsamples of the data set. For each split, a random subset of the available features is used for splitting. The result or final value is determined by majority decisions or averages (Breiman 2001).	Random forest is an effective tool for prediction and is relatively robust against outliers and noise. Random forest reduced the over-fitting problem and usually achieves a good bias-variance tradeoff (Singh et al. 2016).
*k*-Nearest-neighbors	The *k*-nearest-neighbor is a non-parametric algorithm. *k*-Nearest-neighbor approximates the association between independent variables and the continuous outcome by averaging the values of the *k*-nearest neighbors (Sreevalsan-Nair 2020).	*k*-Nearest-neighbor is a simple technique, non-parametric and quick to implement. In addition, the scheme is very flexible (Ray 2019).
Gradient boosting regression	Gradient boosting regression creates a sophisticated model based on a combination of multiple weak individual models, which are mostly decision trees (Sarker 2021; Li et al. 2022).	Boosting is one of the most successful techniques introduced to solve complex problems (Li et al. 2022). Gradient boosting regression is a meaningful and robust model that is suitable for unclean data (Friedman 2001).

### Statistical analysis

All statistical analyses were conducted with Python 3.9 (Van Rossum and Drake 2009) (supplements Table C). First, participant characteristics were presented by descriptive statistics [mean ± standard deviation (SD)]. Two missing values of the Pittsburgh Sleep Quality Index (PSQI) Score were replaced by the single imputation method (Glas 2010). Since the outcome parameters are continuous, the machine learning models were trained for regression tasks. All variables were standardized using the *z* transformation. The final study population was divided into a training (80%) and a validation (20%) set. On the training data, fivefold cross-validation (Refaeilzadeh et al. 2016) was used for hyperparameter tuning using Bayes search (Lindauer et al. 2019) and to train the final models. The performance of the final models was evaluated on the validation set. The evaluation was based on quality criteria including the mean of the reset standardized root mean square error (RMSE), the *R* squared (*R*^2^), the Wasserstein distance (WSD), and the respective 95% confidence intervals obtained from the validation set using 1000 replicates. The Shapley additive explanation (SHAP) (Nohara et al. 2022) was used to determine the feature importance. This involved assessing the relevance of features using SHAP values to identify the relative contribution of the feature to $\dot{V}$O_2peak_ and PPO prediction. In a further step, the entire procedure described above was performed again for $\dot{V}$O_2peak_ and PPO separately for females and males to investigate sex-specific differences. Accordingly, the feature sex was removed from this part of the analysis.

## Results

### Characteristics and CPET values of participants included in the predictions

In total, *n* = 274 potential participants attended the study. Finally, *n* = 258 (♀101, ♂157) adults were included in the analysis for the prediction of $\dot{V}$O_2peak_. Of the *n* = 16 excluded participants, *n* = 14 did not reach a $\dot{V}$O_2_ plateau or at least two out of three exhaustion criteria and *n* = 2 were excluded due to missing BIA and CPET values. In the analysis for predicting PPO, *n* = 272 (♀106, ♂166) participants were included. Only *n* = 2 were excluded due to missing CPET and BIA values. The participants’ characteristics and CPET values are shown in Table 3.

**Table 3:** Participants’ characteristics and CPET values for the predictions (mean ± SD)

Characteristics and CPET values	Prediction of $\dot{V}$O_2peak_			Prediction of PPO		
	Females (*n* = 101)	Males (*n* = 157)	All (*n* = 258)	Females (*n* = 106)	Males (*n* = 166)	All (*n* = 272)
Age (years)	27.40 ± 9.96	27.17 ± 8.46	27.26 ± 9.06	27.29 ± 9.74	27.52 ± 8.97	27.43 ± 9.26
Height (m)	1.69 ± 0.06	1.81 ± 0.07	1.77 ± 0.09	1.69 ± 0.06	1.81 ± 0.07	1.76 ± 0.09
Weight (kg)	65.97 ± 9.60	79.97 ± 10.97	74.49 ± 12.48	65.83 ± 9.52	79.96 ± 10.84	74.45 ± 12.42
BMI	23.09 ± 3.24	24.32 ± 2.98	23.84 ± 3.14	23.03 ± 3.20	24.39 ± 2.99	23.86 ± 3.14
Waist-Hip-Ratio	0.76 ± 0.05	0.83 ± 0.05	0.80 ± 0.06	0.76 ± 0.05	0.83 ± 0.05	0.80 ± 0.06
Rel. FM (%)	27.74 ± 7.60	17.02 ± 6.73	21.22 ± 8.80	27.78 ± 7.53	17.04 ± 6.83	21.23 ± 8.83
Rel. FFM (%)	72.29 ± 7.61	82.98 ± 6.73	78.79 ± 8.80	72.25 ± 7.54	82.96 ± 6.83	78.78 ± 8.82
PPO (W)	214 ± 44	322 ± 57	280 ± 74	212 ± 44	320 ± 60	278 ± 75
$\dot{V}$O_2peak_ (L/min)	2.53 ± 0.51	3.87 ± 0.65	3.34 ± 0.88	2.52 ± 0.51	3.84 ± 0.66	3.33 ± 0.89
$\dot{V}$O_2peak_ (ml/kg/min)	38.89 ± 8.05	48.89 ± 8.34	44.97 ± 9.56	38.71 ± 7.93	48.60 ± 8.56	44.74 ± 9.61
TTE (min:s)	09:41 ± 1:57	10:14 ± 1:49	10:01 ± 1:53	09:37 ± 1:56	10:07 ± 1:55	09:55 ± 1:56

*SD* standard deviation, *CPET* cardiopulmonary exercise testing, *BMI* body-mass-index, *Rel. FM* relative fat mass, *Rel. FFM* relative fat free mass, *PPO* peak power output, $\dot{V}$*O*_*2max*_ maximal oxygen consumption, *TTE* time to exhaustion

### Model comparison for predicting $\dot{V}$O_2peak_ and PPO

**Fig. 1:**
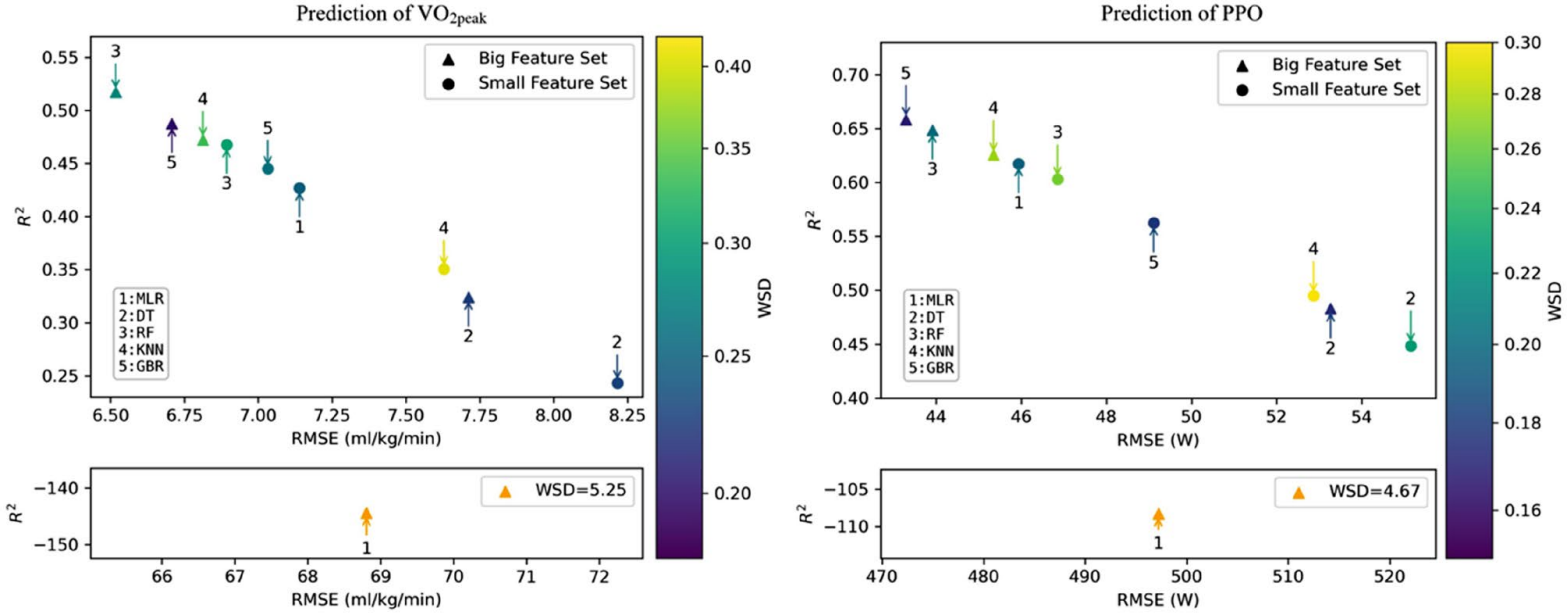
Quality criteria of the models for the prediction of $\dot{V}$O_2peak_ and PPO. *RMSE* root mean squared error, *R*^2^ *R* squared, *WSD* Wasserstein distance, *MLR* multiple linear regression, *DT*: decision tree, *RF* Random forest, *KNN* *k*-nearest-neighbor, *GBR* gradient boosting regression

Figure 1 illustrates the performance of the multiple linear regression and the applied machine learning models concerning the mean of the three quality criteria: RMSE, *R*^2^ and WSD. The RMSE is a standard statistical parameter and is used to evaluate model performance. The units of RMSE in this work are ml/kg/min for the prediction of $\dot{V}$O_2peak_ and W for the prediction of PPO. The *R*^2^ represents the proportion of the variance of the outcome parameter that is explained by the features of the model. The WSD measures differences between probability distributions. The smaller the RMSE and the WSD and the larger the *R*^2^ the more accurate the results of the models. The multiple linear regression with the big feature set has an additional coordinate system, as all quality criteria differ significantly from those of the other models. The mean values of the quality criteria and the 95% confidence intervals can be found in the supplements (Table D–F).

**Fig. 2:**
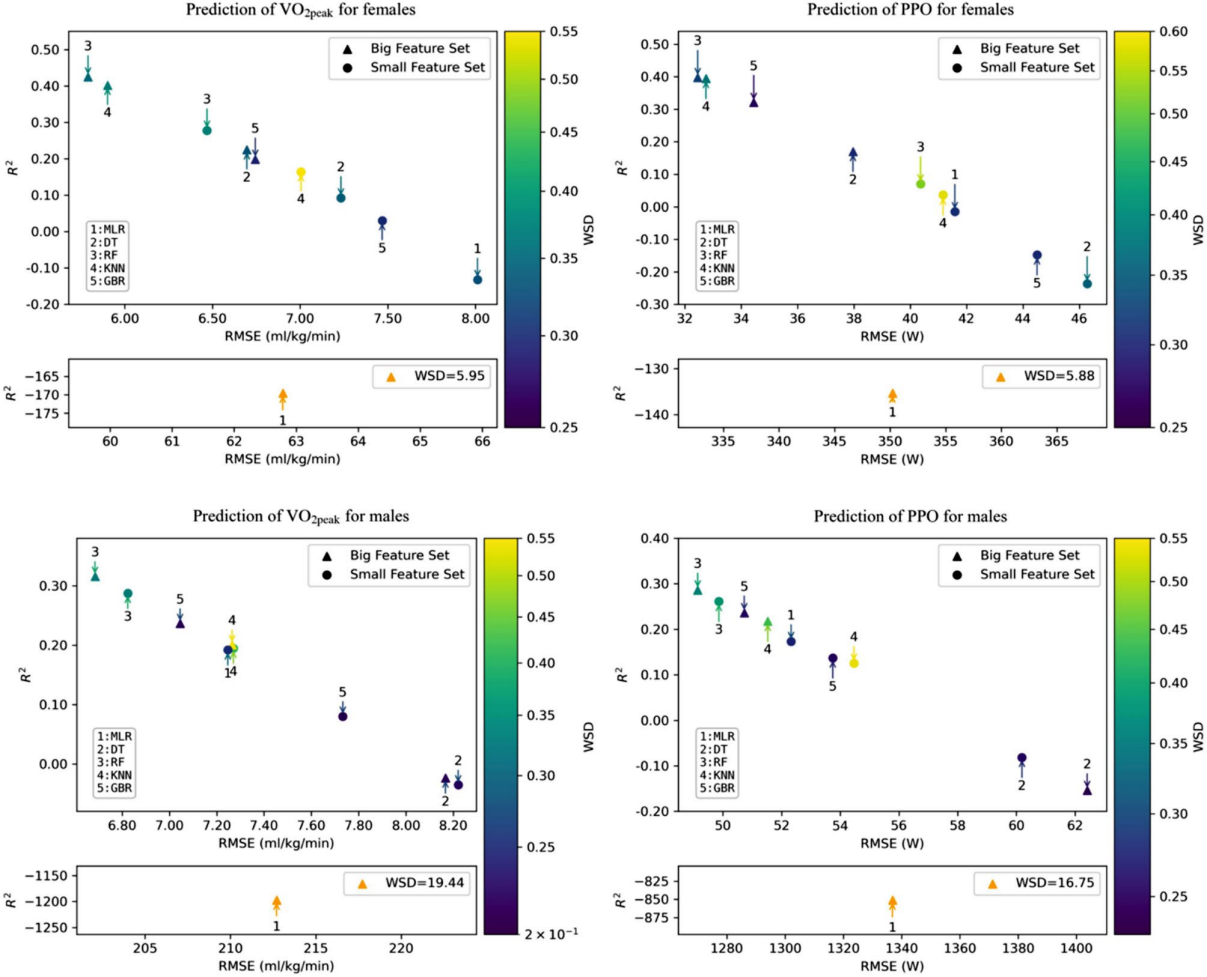
Quality criteria of the models for the prediction of $\dot{V}$O_2peak_ and PPO for females and males. *RMSE* root mean squared error, *R*^2^ *R* squared, *WSD* Wasserstein distance, *MLR* multiple linear regression, *DT* decision tree, *RF* random forest, *KNN* *k*-nearest-neighbor, *GBR* gradient boosting regression

Machine learning models such as random forest and gradient boosting regression with the big feature set have a low RMSE and WSD as well as a high R^2^ and perform better overall than models with the small feature set. Figure 2 shows the performance of the sex-separated models for the prediction of $\dot{V}$O_2peak_ and PPO using the mean of quality criteria.

In the prediction of $\dot{V}$O_2peak_ for females, the applied machine learning models consistently outperformed the multiple linear regression models. In all sex-separated models, the machine learning models with the big feature set perform better overall than the models with the small set.

### Feature importance

**Fig. 3:**
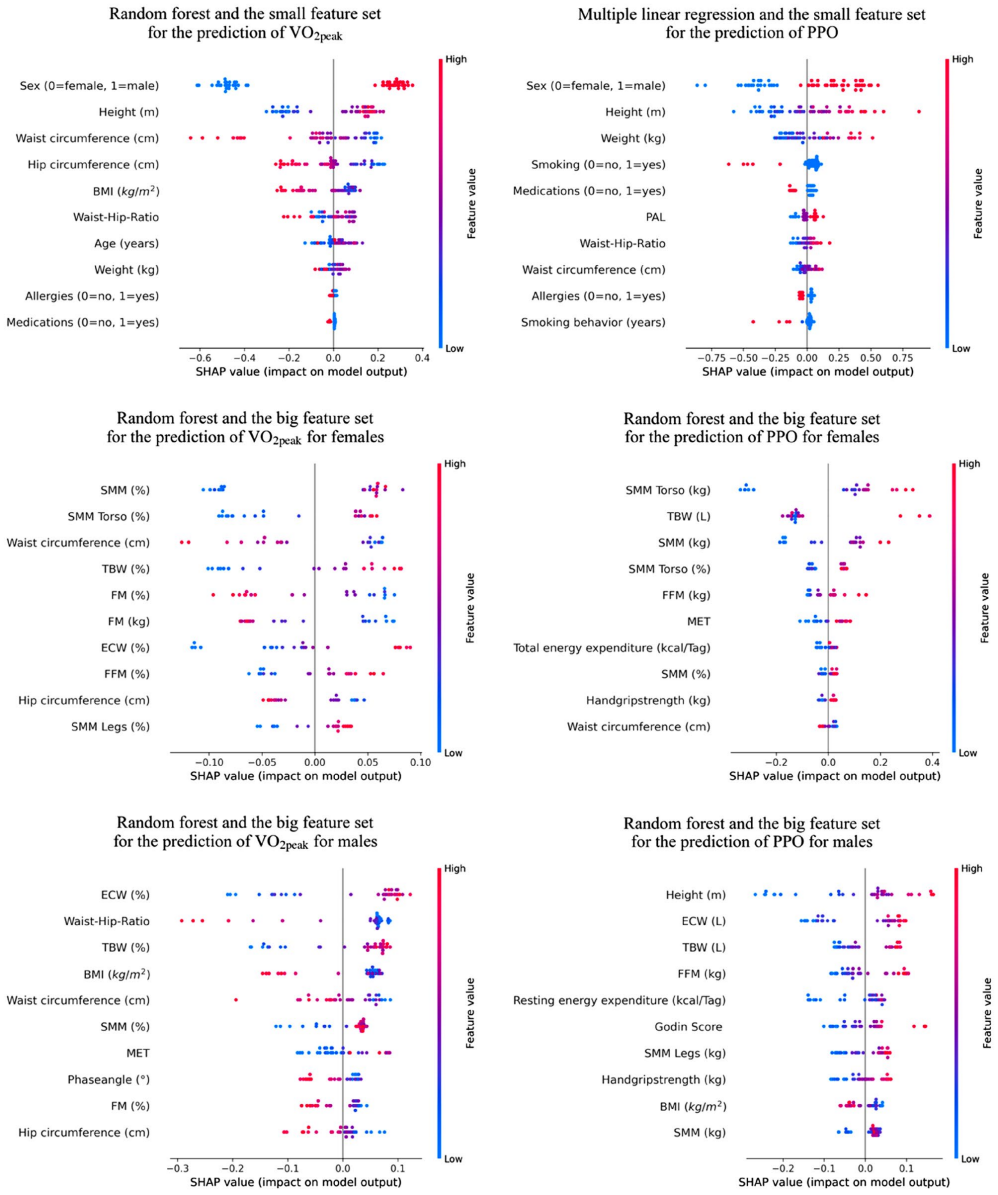
Importance of the features by SHAP with the random forest and multiple linear regression with the small feature set as well as the random forest with the big feature set for the prediction of $\dot{V}$O_2peak_ and PPO for females and males. *SHAP* Shapley additive explanation, *BMI* body-mass-index, *PAL* physical activity level, *SMM* skeletal muscle mass, *TBW* total body water, *FM* fat mass, *FFM* fat free mass, *ECW* extracellular water, *MET* metabolic equivalent task

The ten most impactful features for predicting $\dot{V}$O_2peak_ and PPO were selected for each model by SHAP. SHAP values are assigned to each feature for prediction. The prediction result is the sum of the contributions of each feature. The *x*-axis represents the impact of each feature on the prediction for each participant represented by a dot and the *y*-axis shows the feature in descending order of overall importance. The color of the gradient denotes the magnitude of the original value for that feature. Since random forest and multiple linear regression performed best among the machine learning models with the small feature set (Fig. 1) and random forest with the big feature set consistently performed best among the sex- separated models, the SHAP values of these models are shown in Fig. 3. The supplements also contain all figures of the SHAP values of the most accurate model with the big and the small feature set (Figure A–F).

## Discussion

We hypothesized that machine learning models will provide more accurate predictions of $\dot{V}$O_2peak_ and PPO than the conventional multiple linear regression technique. Moreover, we aimed (1) to compare two non-exercise feature sets to predict $\dot{V}$O_2peak_ and PPO using four machine learning models as well as multiple linear regression and (2) to identify the most impactful features. The results confirm that machine learning models provide more precise results in comparison to multiple linear regression. Our analysis further indicates that machine learning models with comprehensive features make more accurate predictions than models containing only anthropometric and demographic data. In particular, features that include information about the participant’s body composition seem to have a relevant impact on the prediction of $\dot{V}$O_2peak_ and PPO. These results may be helpful in developing new standards for performing CPETs and improving prediction models for $\dot{V}$O_2peak_ and PPO.

In addition to the four machine learning models decision tree, random forest, *k*-nearest-neighbor and gradient boosting regression, we used multiple linear regression as a conventional technique that is commonly used in exercise physiology to predict $\dot{V}$O_2peak_ and PPO (Myers et al. 1994, 2001; da Silva et al. 2012; Akay and Abut 2015; Saengsuwan et al. 2017). Previous literature has already shown that intelligent machine learning models can predict $\dot{V}$O_2peak_ more accurately than existing multiple linear regression-based prediction models (Akay and Abut 2015; Liu et al. 2022). These results are confirmed by our work, which, in contrast to previous literature, compared machine learning and multiple linear regression models based on the same population and the same conditions. Moreover, these indicate that some predictor variables showed non-linear relationships with $\dot{V}$O_2peak_ and PPO. The applied machine learning models can effectively analyze and capture these non-linear relations, explaining their greater performance over the traditional multiple linear regression technique.

In this study, the random forest proved to be the most robust prediction model, as it possessed the lowest RMSE, the highest *R*^2^, and typically displayed a lower WSD for almost all predictions. The random forest estimated $\dot{V}$O_2peak_ considering both sexes with a mean error of 6.52 ml/kg/min, a variance explanation of approximately 52% and a difference in probability distributions of 0.28. The gradient boosting regression model performed slightly better than the random forest in predicting PPO when both sexes were considered. It predicted PPO with a mean error of 43 W, a variance explanation of about 66% and a difference in the probability distributions of 0.18. In particular, the multiple linear regression had an unusually high RMSE and WSD as well as a negative *R*^2^ if many features were included in the prediction. The results were outside the interpretable range and are due to the fact that the multiple linear regression cannot handle a large number of predictor variables that exhibit multicollinearity (Jobson 1991). In contrast to multiple linear regression, random forest handles outliers and avoids overfitting by capturing underlying patterns rather than overlearning the training data (Singh et al. 2016). The gradient boosting regression is considered a robust method that can also deal with very heterogeneous data (Friedman 2001). In addition to these advantages, machine learning models are able to recognize linear relationships between variables. Therefore, the effectiveness of conventional linear methods in predicting $\dot{V}$O_2peak_ and PPO should be critically reconsidered.

Beside the correct selection of suitable prediction models, the identification of relevant features is crucial. This allows practical recommendations regarding the parameters that should be recorded before conducting a CPET. Previous literature aimed at individualizing test protocols has used features that are usually assessed prior CPET, such as questionnaires on PAL, sex, age, BMI or resting HR (Myers et al. 2001; da Silva et al. 2012; Cunha et al. 2015; Saengsuwan et al. 2017). In our study, we divided the features into two sets to determine if collecting only anthropometric and demographic data before conducting a CPET is sufficient to adapt a ramp protocol to the participant’s characteristics.

Previously, data from the National Health and Nutrition Examination Survey (Liu et al. 2022) have been used to develop machine learning models for the prediction of $\dot{V}$O_2max_ with non-exercise features. In line with our results, the authors concluded that models with a comprehensive feature set performed significantly better than previous methods using a limited number of predictors and mainly linear models. However, the work was limited by the fact that some predictor variables cannot be readily implemented in other healthcare settings. Furthermore, existing studies have often used submaximal features to predict maximal physical performance, which are less practical and more time-consuming (Evans et al. 2015; Kokkinos et al. 2018; Abut et al. 2019; Ashfaq et al. 2022). In our work, only non-exercise features that are convenient to collect in various environments were included.

To elaborate which features contribute most to the prediction of $\dot{V}$O_2peak_ and PPO, the results of the SHAP analysis were considered. As in previous studies (Myers et al. 2001; da Silva et al. 2012), our findings indicate that sex has a significant influence on the prediction when the small feature set is applied. To determine what accounts for the difference between males and females, we fitted the models to the sex-separated data and examined the big feature set in the following.

Body composition variables proved to be the most influential features of $\dot{V}$O_2peak_ and PPO. Especially SMM, ECW, and TBW seem to be important predictors. The SHAP analysis showed that high body composition values associated with high SMM led to increased $\dot{V}$O_2peak_ and PPO. This can be explained by the fact that muscle fibers consume oxygen and fiber cross-sectional areas increase linearly with PPO (Appelman et al. 2024). Furthermore, there exists a linear relationship between the power output and the $\dot{V}$O_2_ increment rate.

Subjective features such as health-related questionnaires, as well as demographic data appeared to be less relevant, particularly for the prediction of $\dot{V}$O_2peak_ for females and PPO for both sexes. The SHAP values for the prediction of $\dot{V}$O_2peak_ and PPO in males attribute a relevant significance to the waist–hip ratio and height. This indicates that anthropometric data should continue to be used for the predictions and should not be completely excluded.

The SHAP analysis showed that it may be beneficial to determine the body composition of the participant before conducting a CPET to adapt a ramp protocol on the cycle ergometer to the characteristics of the participant. With the prediction of PPO, an adaptation of the protocol can be implemented quickly. To effectively utilize the predicted $\dot{V}$O_2peak_ from this work, the $\dot{V}$O_2_ and power output relationship can be considered, which is approximately 10 ml/W/min. The mean response time of $\dot{V}$O_2_ for ramp protocols is about 40 s (Caen et al. 2020). Using these two variables, the rate of power increase required to reach the predicted $\dot{V}$O_2peak_ in a given time can be calculated.

A limiting factor of this work is that the PPO is influenced by the choice of power increase (Poole and Jones 2017). This reduces the reliability of the models for predicting PPO as they are based on the ramp protocols performed in this study. Consequently, we included $\dot{V}$O_2peak_ as an outcome parameter since it can be achieved despite different power output slopes (Iannetta et al. 2020). Moreover, the generalizability of the prediction models is limited to the investigated population, comprising mainly healthy young European adults who were physically able to perform a CPET.

The results can help to adjust power increase in a ramp protocol to achieve volitional exhaustion within a certain duration. This facilitates the comparison of CPETs between different test settings, clinical environments, and studies. In addition, the results can be used to evaluate the effectiveness of an intervention to increase PPO or $\dot{V}$O_2peak_. This involves adapting the power increase in a CPET before and after the intervention using the machine learning prediction models. The results could be used to assess an individual’s exercise tolerance by using the machine learning prediction models to determine when an individual's measured $\dot{V}$O_2peak_ is significantly different from the predicted values. In future analysis, the machine learning approach can be extended to predictions for clinical populations by adding disease-specific features. In addition, the population can be extended to a wider age range, different body mass classes and lower fitness levels.

## Supplementary Information

Below is the link to the electronic supplementary material.

Supplementary file1 (DOCX 528 KB)

## Data Availability

Data are available on reasonable request.

## Abbreviations

BIA: Bioelectrical impedance analysis
BMI: Body-mass-index
CPET: Cardiopulmonary exercise testing
ECW: Extracellular water
FFM: Fat free mass
FM: Fat mass
HR: Heart rate
HR_max_: Maximal heart rate
MET: Metabolic equivalent task
PAL: Physical activity level
PPO: Peak power output
PSQI: Pittsburgh Sleep Quality Index
RER: Respiratory exchange ratio
RMSE: Root mean square error
SD: Standard deviation
SHAP: Shapley additive explanation
SMM: Skeletal muscle mass
TBW: Total body water
TTE: Time to exhaustion
$\dot{V}$O_2max_: Maximal oxygen uptake
$\dot{V}$O_2peak_: Peak oxygen uptake
W: Watts
WSD: Wasserstein distance
