# Supplementary material for: Machine learning predicts peak oxygen uptake and peak power output for customizing cardiopulmonary exercise testing using non-exercise features
Source: Eur J Appl Physiol. 2024 Jul 3;124(11):3421–31. doi: 10.1007/s00421-024-05543-x (PMC11519113; doi:10.1007/s00421-024-05543-x)
Supplement: Supplementary file 1 — Supplementary file1 (DOCX 528 KB) [file 421_2024_5543_MOESM1_ESM.docx]

**European Journal of Applied Physiology**

**Machine learning predicts peak oxygen uptake and peak power output for customizing cardiopulmonary exercise testing using non-exercise features**

Charlotte Wenzel^1^, Thomas Liebig^2^, Adrian Swoboda^3^, Rika Smolareck^1^, Marit L Schlagheck^1^, David Walzik^1^, Andreas Groll^4^, Richie P Goulding^5^, Philipp Zimmer^1*^.

^1^ Institute for Sport and Sport Science, Performance and Health (Sports Medicine), TU Dortmund University, Dortmund, Germany

^2^ Institute for Computer Science, Department of Artificial Intelligence, TU Dortmund University, Dortmund, Germany

^3^ Institute for Training Optimization for Sport and Health, iQ athletik, Frankfurt am Main, Germany

^4^ Department of Statistics, Statistical Methods for Big Data, TU Dortmund University, Dortmund, Germany

^5^ Faculty of Behavioral and Movement Sciences, Department of Human Movement Sciences, Vrije Universiteit Amsterdam, Amsterdam Movement Sciences, Amsterdam, the Netherlands

^*^Corresponding author, Institute for Sport and Sport Science, Performance and Health (Sports Medicine), TU Dortmund University, Otto- Hahn-Str. 3, 44227 Dortmund, Germany, Tel.: +49 231 755 7436, Dortmund, Germany; [philipp.zimmer@tu-dortmund.de](mailto:philipp.zimmer@tu-dortmund.de)

**Supplements**

Table A. Increase of the ramp protocols

|  | **BMI and PAL category** | **Power increase (W/min)** |
| --- | --- | --- |
| **Females** | ≥ 25 or < 20 and low | 15 |
|  | All other cases | 20 |
|  | ≥ 25 or < 20 and high | 25 |
| **Males** | ≥ 25 or < 20 and low | 20 |
|  | All other cases | 25 |
|  | ≥ 25 or < 20 and high | 30 |

BMI: Body-Mass-Index; PAL: Physical activity level.

Table B. All collected features.

| **Feature** | **Section** | **Data type** | **Method** | **Definition** |
| --- | --- | --- | --- | --- |
| Age | Demographic | Continuous | In-person interview | Years of the participant at time of the study |
| Sex | Demographic | Binary | In-person interview | Coded as:  0 = female  1 = male |
| Smoking | Questionnaire | Binary | In-person interview | Coded as:  0 = non-smoker  1 = smoker |
| Smoking behaviour | Questionnaire | Continuous | In-person interview | Years of active Smoking |
| Chronic disease | Questionnaire | Binary | In-person interview | Coded as:  0 = no chronic disease  1 = chronic disease |
| Allergies | Questionnaire | Binary | In-person interview | Coded as:  0 = no allergies  1 = allergies |
| Supplements | Questionnaire | Binary | In-person interview | Coded as:  0 = no supplements  1 = taking supplements |
| Medication | Questionnaire | Binary | In-person interview | Coded as:  0 = no medication  1 = taking medication |
| Physical Activity Level (PAL) | Questionnaire | Categorical | In-person interview | Code as:  0 = almost exclusively lying  1 = almost exclusively sitting 2 = predominantly sitting, occasionally standing 3 = predominantly standing or walking  4 = physically demanding |
| Weight | Examination | Continuous | Measured with Scale MPE Kern | Weight in kg |
| Height | Examination | Continuous | Measured with MPE Kern | Height in cm |
| Body-Mass-Index (BMI) | Examination | Continuous | Calculated with height and weight | Calculated by: kg/m^2^ |
| Waist circumference | Examination | Continuous | Measured with circumference tape (SECA) | Waist circumference in cm |
| Hip circumference | Examination | Continuous | Measured with circumference tape (SECA) | Hip circumference in cm |
| Waist-Hip-Ratio | Examination | Continuous | Calculated with Waist circumference and Hip circumference | Calculated by:  Waist circumference (cm) / Hip circumference (cm) |
| Relative fat mass (FM) | Examination | Continuous | Measured with bioelectric impedance analysis (SECA mBCA 525) | Fat mass in % |
| Absolute fat mass (FM) | Examination | Continuous | Measured with bioelectric impedance analysis (SECA mBCA 525) | Fat mass in kg |
| Relative Fat free mass (FFM) | Examination | Continuous | Measured with bioelectric impedance analysis (SECA mBCA 525) | Fat free mass in % |
| Absolute fat free mass (FFM) | Examination | Continuous | Measured with bioelectric impedance analysis (SECA mBCA 525) | Fat free mass in kg |
| Relative skeletal muscle mass (SMM) | Examination | Continuous | Measured with bioelectric impedance analysis (SECA mBCA 525) | Skeletal muscle mass in % |
| Absolute skeletal muscle mass (SMM) | Examination | Continuous | Measured with bioelectric impedance analysis (SECA mBCA 525) | Skeletal muscle mass in kg |
| Relative skeletal muscle mass (SMM) torso | Examination | Continuous | Measured with bioelectric impedance analysis (SECA mBCA 525) | Skeletal muscle mass Torso in % |
| Absolute skeletal muscle mass (SMM) torso | Examination | Continuous | Measured with bioelectric impedance analysis (SECA mBCA 525) | Skeletal muscle mass torso in kg |
| Relative skeletal muscle mass (SMM) legs | Examination | Continuous | Measured with bioelectric impedance analysis (SECA mBCA 525) | Skeletal muscle mass legs in % |
| Absolute skeletal muscle mass (SMM) legs | Examination | Continuous | Measured with bioelectric impedance analysis (SECA mBCA 525) | Skeletal muscle mass legs in kg |
| Relative total body water (TBW) | Examination | Continuous | Measured with bioelectric impedance analysis (SECA mBCA 525) | Total body water in % |
| Absolute total body water (TBW) | Examination | Continuous | Measured with bioelectric impedance analysis (SECA mBCA 525) | Total body water in l |
| Relative extracellular water (ECW) | Examination | Continuous | Measured with bioelectric impedance analysis (SECA mBCA 525) | Extracellular water in % |
| Absolute extracellular water (ECW) | Examination | Continuous | Measured with bioelectric impedance analysis (SECA mBCA 525) | Extracellular water in l |
| Ratio of extracellular water and total body water (ECW/TBW) | Examination | Continuous | Measured with bioelectric impedance analysis (SECA mBCA 525) | Calculated by: extracellular water (l)/ total body water (l) |
| Total energy expenditure | Examination | Continuous | Measured with bioelectric impedance analysis (SECA mBCA 525) | Total energy expenditure in kcal/day |
| Resting energy expenditure | Examination | Continuous | Measured with bioelectric impedance analysis (SECA mBCA 525) | Resting energy expenditure in kcal/day |
| Phase angle | Examination | Continuous | Measured with bioelectric impedance analysis (SECA mBCA 525) | Phase angle in ° (percentile) |
| Relative handgrip strength (mean) | Examination | Continuous | Measured with handgrip force dynamometer | Mean handgrip strength of the left and right hand together in % |
| Absolute handgrip strength (mean) | Examination | Continuous | Measured with handgrip force dynamometer | Mean handgrip strength of the left and right hand together in kg |
| Metabolic equivalent task (MET) | Questionnaire | Continuous | In-person interview  Official calculation of Global physical activity questionnaire (GPAQ) |  |
| Physical activity level (PAL) category | Questionnaire | Categorical | In-person interview  Official calculation of Global physical activity questionnaire (GPAQ) | Coded as:  0 = Low  1 = Moderate  2 = High |
| Godin Score | Questionnaire | Continuous | Independent questionnaire filling  Official calculation of Godin Leisure-time Exercise (GODIN) Questionnaire |  |
| Godin category | Questionnaire | Categorical | Independent questionnaire filling  Official calculation of Godin Leisure-time Exercise (GODIN) Questionnaire | Coded as:  0 = Insufficiently active  1 = Moderately active  2 = Active |
| Pittsburgh Sleep Quality Index (PSQI) Score | Questionnaire | Continuous | Independent questionnaire filling  Official Calculation of Pittsburgh Sleep Quality Index (PSQI) Questionnaire | Range from 0 to 21 |
| Pittsburgh Sleep Quality Index (PSQI) category | Questionnaire | Categorical | Independent questionnaire filling  Official Calculation of Pittsburgh Sleep Quality Index (PSQI) Questionnaire | Coded as:  0 = Chronical sleep disorder  1 = Unhealthy sleep  2 = Healthy sleep |

Table C. Applied python packages.

| **Packages** | **Version** |
| --- | --- |
| Pandas | 2.1.4 |
| Numpy | 1.26.2 |
| Matplotlib | 3.8.2 |
| Seaborn | 0.13.0 |
| SciPy | 1.11.4 |
| Shap | 0.44.0 |
| Scikit-learn | 1.3.2 |
| Papermill | 2.4.0 |

Table D. Quality criteria of the models for the prediction of V̇O_2peak_ and PPO (mean [95%CI]).

| **Model** | **Feature set** | **RMSE** | | **R^2^** | | **WSD** | |
| --- | --- | --- | --- | --- | --- | --- | --- |
|  | | **PPO** | **V̇O_2peak_** | **PPO** | **V̇O_2peak_** | **PPO** | **V̇O_2peak_** |
| **MLR** | small | **45.94** (W) [95%CI: 38.21- 54.36] | 7.14 (ml/kg/min) [95%CI: 5.69- 8.84] | **0.62** [95%CI: 0.48-0.73] | 0.43 [95%CI: 0.18-0.62] | 0.20 [95%CI: 0.13 -0.28] | 0.25 [95%CI: 0.14-0.36] |
|  | big | 497.21 (W) [95%CI: 51.58 -2656.29] | 68.81 (ml/kg/min) [95%CI: 7.16- 343.48] | -108.29 [95%CI: -1246.29 -0.56] | -144.45 [95%CI: -1311.38- 0.41] | 4.67 [95%CI: 0.20 -26.00] | 5.25 [95%CI: 0.19- 28.84] |
| **DT** | small | 55.15 (W) [95%CI: 45.11- 66.23] | 8.22 (ml/kg/min) [95%CI: 6.45- 10.22] | 0.45 [95%CI: 0.24-0.62] | 0.24 [95%CI: -0.06-0.49] | 0.23 [95%CI: 0.16-0.34] | **0.23** [95%CI: 0.15 -0.35] |
|  | big | 53.27 (W) [95%CI: 42.71- 65.33] | 7.71 (ml/kg/min) [95%CI: 5.98- 9.73] | 0.48 [95%CI: 0.27 -0.65] | 0.32 [95%CI: 0.04-0.55] | 0.18 [95%CI: 0.12 -0.26] | 0.23 [95%CI: 0.17- 0.31] |
| **RF** | small | 46.85 (W) [95%CI: 39.56- 54.74] | **6.89** (ml/kg/min) [95%CI: 5.58- 8.40] | 0.60 [95%CI: 0.47 -0.72] | **0.47** [95%CI: 0.28-0.62] | 0.27 [95%CI: 0.21 -0.33] | 0.30 [95%CI: 0.22-0.40] |
|  | big | 43.91 (W) [95%CI: 36.33- 52.60] | **6.52** (ml/kg/min) [95%CI: 5.21- 8.17] | 0.65 [95%CI: 0.52 -0.76] | **0.52** [95%CI: 0.32- 0.66] | 0.21 [95%CI: 0.15 -0.28] | 0.28 [95%CI: 0.22- 0.35] |
| **KNN** | small | 52.87 (W) [95%CI: 43.02- 63.29] | 7.63 (ml/kg/min) [95%CI: 6.17- 9.29] | 0.49 [95%CI: 0.32 -0.65] | 0.35 [95%CI: 0.17-0.51] | 0.30 [95%CI: 0.22 -0.40] | 0.41 [95%CI: 0.32 -0.50] |
|  | big | 45.35 (W) [95%CI: 37.02- 53.91] | 6.81 (ml/kg/min) [95%CI: 5.49- 8.54] | 0.63 [95%CI: 0.51- 0.71] | 0.47 [95%CI: 0.26- 0.63] | 0.28 [95%CI: 0.20 -0.36] | 0.34 [95%CI: 0.24- 0.43] |
| **GBR** | small | 49.11 (W) [95%CI: 39.82- 58.72] | 7.03 (ml/kg/min) [95%CI: 5.72- 8.63] | 0.56 [95%CI: 0.40 -0.71] | 0.44 [95%CI: 0.23 -0.62] | **0.19** [95%CI: 0.12 -0.27] | 0.26 [95%CI: 0.18-0.36] |
|  | big | **43.29** (W) [95%CI: 35.11- 52.34] | 6.71 (ml/kg/min) [95%CI: 5.18- 8.55] | **0.66** [95%CI: 0.53- 0.78] | 0.49 [95%CI: 0.25- 0.66] | **0.18** [95%CI: 0.12 -0.25] | **0.20** [95%CI: 0.13- 0.29] |

CI: Confidence interval; RMSE: Root mean squared error; 𝑅^2^: R squared; WSD: Wasserstein distance; MLR: Multiple linear regression; DT: Decision tree; RF: Random forest; KNN: k-nearest-neighbor; GBR: Gradient boosting regression; W: Watts.

Table E. Quality criteria of the models for the prediction of V̇O_2peak_ and PPO for females (mean [95% CI]).

| Model | **Feature set** | **RMSE** | | **R^2^** | | **WSD** | |
| --- | --- | --- | --- | --- | --- | --- | --- |
|  | | **PPO** | **V̇O_2peak_** | **PPO** | **V̇O_2peak_** | **PPO** | **V̇O_2peak_** |
| MLR | small | 41.58 (W) [95%CI: 28.11- 59.36] | 8.01 (ml/kg/min) [95%CI: 4.77- 13.29] | -0.01 [95%CI: -0.86- 0.43] | -0.13 [95%CI: -1.14- 0.43] | 0.32 [95%CI: 0.16- 0.51] | 0.34 [95%CI: 0.17- 0.56] |
|  | big | 350.20 (W) [95%CI: 56.34- 1454.45] | 62.78 (ml/kg/min) [95%CI: 6.86- 283.23] | -135.32 [95%CI: -998.20- -0.90] | -169.58 [95%CI: -1581.64- -0.03] | 5.88 [95%CI: 0.30 -25.55] | 5.95 [95%CI: 0.24 -31.82] |
| DT | small | 46.28 (W) [95%CI: 31.91- 63.54] | 7.23 (ml/kg/min) [95%CI: 4.65- 11.06] | -0.24 [95%CI: -0.83- 0.26] | 0.09 [95%CI: -0.40 -0.50] | 0.37 [95%CI: 0.24- 0.54] | 0.35 [95%CI: 0.24- 0.50] |
|  | big | 37.96 (W) [95%CI: 25.27- 52.60] | 6.70 (ml/kg/min) [95%CI: 3.97- 10.63] | 0.17 [95%CI: -0.40- 0.59] | 0.22 [95%CI: -0.23- 0.56] | 0.31 [95%CI: 0.20 -0.45] | 0.34 [95%CI: 0.24 -0.51] |
| RF | small | **40.36** (W) [95%CI: 29.33- 52.74] | **6.47** (ml/kg/min) [95%CI: 4.47- 9.33] | **0.07** [95%CI: -0.18 - 0.26] | **0.28** [95%CI: 0.00- 0.48] | 0.56 [95%CI: 0.44- 0.66] | 0.39 [95%CI: 0.30- 0.51] |
|  | big | **32.45** (W) [95%CI: 23.20- 43.99] | **5.79** (ml/kg/min) [95%CI: 3.61- 8.93] | **0.40** [95%CI: 0.02- 0.65] | **0.42** [95%CI: 0.19- 0.61] | 0.33 [95%CI: 0.24- 0.45] | 0.37 [95%CI: 0.29- 0.45] |
| KNN | small | 41.16 (W) [95%CI: 29.07- 54.40] | 7.01 (ml/kg/min) [95%CI: 4.71- 10.22] | 0.04 [95%CI: -0.16- 0.21] | 0.16 [95%CI: 0.01- 0.29] | 0.63 [95%CI: 0.52- 0.73] | 0.60 [95%CI: 0.48- 0.70] |
|  | big | 32.75 (W) [95%CI: 23.96- 43.18] | 5.90 (ml/kg/min) [95%CI: 3.74- 8.86] | 0.39 [95%CI: 0.13- 0.58] | 0.40 [95%CI: 0.18 -0.59] | 0.39 [95%CI: 0.29- 0.52] | 0.39 [95%CI: 0.28- 0.49] |
| GBR | small | 44.50 (W) [95%CI: 30.21- 61.46] | 7.47 (ml/kg/min) [95%CI: 4.76- 11.51] | -0.15 [95%CI: -0.73- 0.35] | 0.03 [95%CI: -0.54- 0.48] | **0.31** [95%CI: 0.18- 0.46] | **0.31** [95%CI: 0.18 -0.46] |
|  | big | 34.44 (W) [95%CI: 23.77- 46.51] | 6.74 (ml/kg/min) [95%CI: 3.79- 10.53] | 0.32 [95%CI: -0.10- 0.64] | 0.20 [95%CI: -0.51- 0.59] | **0.27** [95%CI: 0.17- 0.40] | **0.30** [95%CI: 0.18- 0.47] |

CI: Confidence interval; RMSE: Root mean squared error; 𝑅^2^: R squared; WSD: Wasserstein distance; MLR: Multiple linear regression; DT: Decision tree; RF: Random forest; KNN: k-nearest-neighbor; GBR: Gradient boosting regression; W: Watts.

Table F. Quality criteria of the models for the prediction of V̇O_2peal_ and PPO for males (mean [95%CI]).

| **Model** | **Feature set** | **RMSE** | | **R^2^** | | **WSD** | |
| --- | --- | --- | --- | --- | --- | --- | --- |
|  | | **PPO** | **V̇O_2peak_** | **PPO** | **V̇O_2peak_** | **PPO** | **V̇O_2peak_** |
| **MLR** | small | 52.32 (W) [95%CI: 40.96- 69.50] | 7.25 (ml/kg/min) [95%CI: 5.44- 9.46] | 0.17 [95%CI: -0.39- 0.47] | 0.19 [95%CI: -0.24- 0.51] | 0.30 [95%CI: 0.15- 0.44] | 0.31 [95%CI: 0.15- 0.47] |
|  | big | 1336.84 (W) [95%CI: 90.56- 4357.66] | 212.70 (ml/kg/min) [95%CI: 13.59- 662.14] | -851.09 [95%CI: -4788.15- -1.47] | -1197.35 [95%CI: -6754.92- -1.92] | 16.75 [95%CI: 0.35- 52.44] | 19.44 [95%CI: 0.53- 62.73] |
| **DT** | small | 60.19 (W) [95%CI: 45.28- 77.42] | 8.22 (ml/kg/min) [95%CI: 5.94- 10.51] | -0.08 [95%CI: -0.61- 0.33] | -0.04 [95%CI: -0.55- 0.36] | 0.28 [95%CI: 0.17- 0.41] | 0.27 [95%CI: 0.18- 0.40] |
|  | big | 62.41 (W) [95%CI: 46.96- 79.29] | 8.17 (ml/kg/min) [95%CI: 5.97- 10.36] | -0.15 [95%CI: -0.65- 0.27] | -0.02 [95%CI: -0.51- 0.40] | **0.26** [95%CI: 0.16- 0.40] | **0.27** [95%CI: 0.18- 0.39] |
| **RF** | small | **49.86** (W) [95%CI: 39.53- 61.00] | **6.82** (ml/kg/min) [95%CI: 5.31- 8.40] | **0.26** [95%CI: -0.02- 0.46] | **0.29** [95%CI: -0.06- 0.50] | 0.42 [95%CI: 0.32- 0.52] | 0.39 [95%CI: 0.31- 0.48] |
|  | big | **49.14** (W) [95%CI: 39.50- 59.29] | **6.69** (ml/kg/min) [95%CI: 5.22- 8.12] | **0.29** [95%CI: 0.03- 0.47] | **0.32** [95%CI: 0.04- 0.52] | 0.39 [95%CI: 0.31- 0.49] | 0.38 [95%CI: 0.30- 0.48] |
| **KNN** | small | 54.46 (W) [95%CI: 43.80- 66.03] | 7.27 (ml/kg/min) [95%CI: 5.71- 9.01] | 0.13 [95%CI: -0.04- 0.26] | 0.19 [95%CI: -0.11- 0.41] | 0.59 [95%CI: 0.51- 0.67] | 0.47 [95%CI: 0.37- 0.57] |
|  | big | 51.53 (W) [95%CI: 41.33- 62.16] | 7.26 (ml/kg/min) [95%CI: 5.83- 8.76] | 0.22 [95%CI: -0.01- 0.41] | 0.20 [95%CI: 0.03- 0.34] | 0.48 [95%CI: 0.39- 0.58] | 0.60 [95%CI: 0.51- 0.67] |
| **GBR** | small | 53.74 (W) [95%CI: 40.00- 68.37] | 7.73 (ml/kg/min) [95%CI: 5.75- 9.88] | 0.14 [95%CI: -0.26- 0.47] | 0.08 [95%CI: -0.41- 0.45] | **0.27** [95%CI: 0.17- 0.40] | **0.26** [95%CI: 0.16- 0.39] |
|  | big | 50.72 (W) [95%CI: 38.70- 64.04] | 7.06 (ml/kg/min) [95%CI: 5.19- 8.89] | 0.24 [95%CI: -0.09- 0.51] | 0.24 [95%CI: -0.14- 0.54] | 0.28 [95%CI: 0.17- 0.40] | 0.27 [95%CI: 0.17- 0.38] |

CI: Confidence interval; RMSE: Root mean squared error; 𝑅^2^: R squared; WSD: Wasserstein distance; MLR: Multiple linear regression; DT: Decision tree; RF: Random forest; KNN: k-nearest-neighbor; GBR: Gradient boosting regression; W: Watts.

| 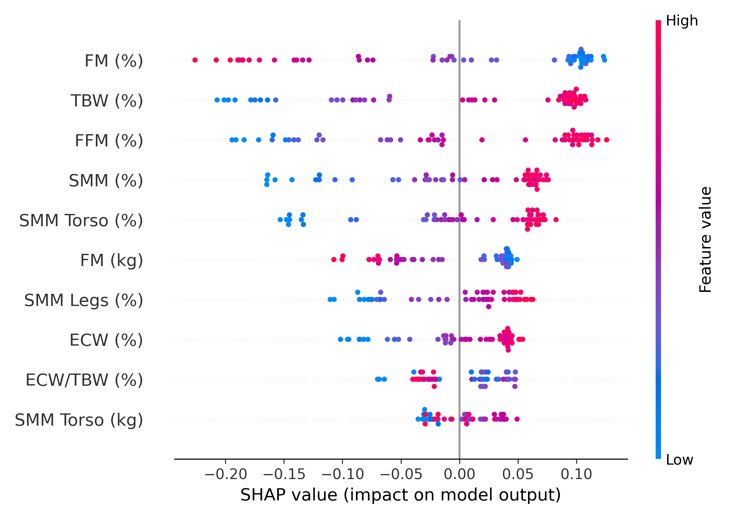 | 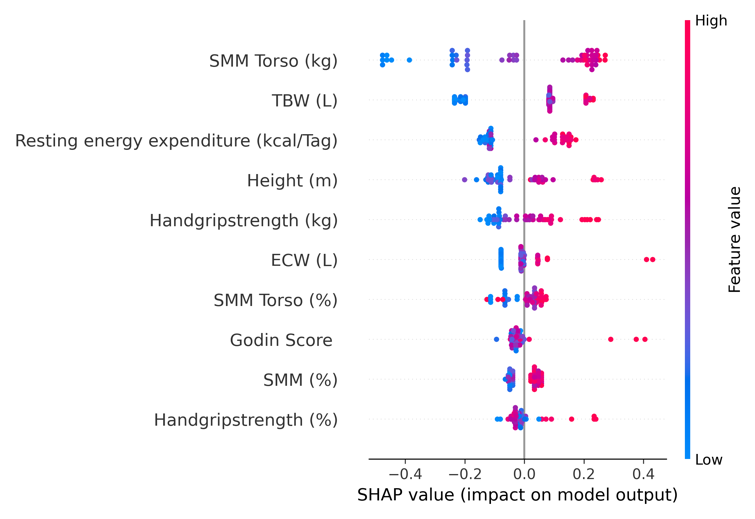 |
| --- | --- |
| **Figure A.** Importance of the features by SHAP with the random forest and the big feature set for the prediction of V̇O_2peak_. FFM: Fat free mass; TBW: Total body water; FM: Fat mass; ECW: Extracellular water; SMM: Skeletal muscle mass. | **Figure B.** Importance of the features by SHAP with the gradient boosting regression and the big feature set for the prediction of PPO_._ TBW: Total body water; FFM: Fat free mass; SMM: Skeletal muscle mass; ECW: Extracellular water. |
| 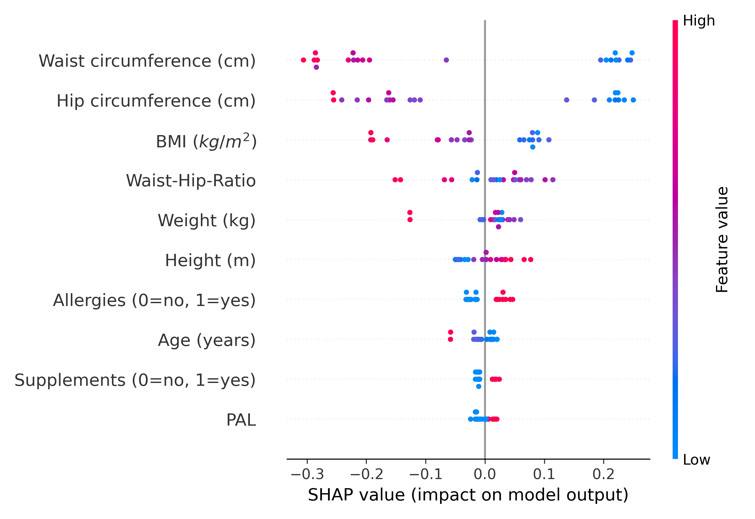 | 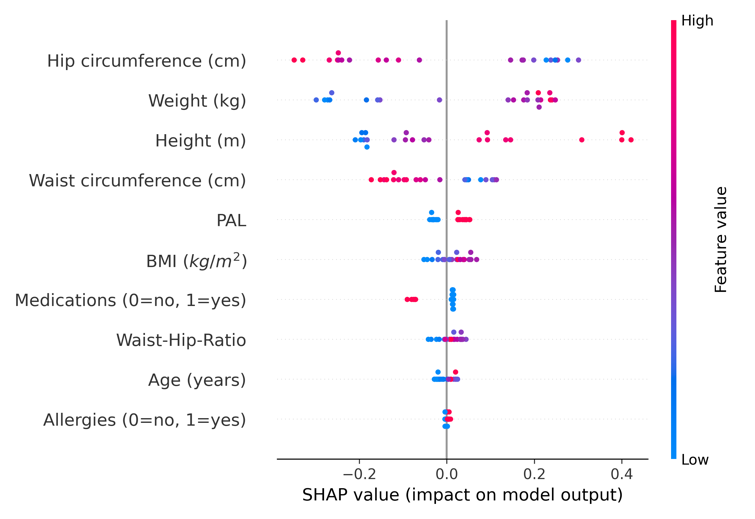 |
| **Figure C.** Importance of the features by SHAP with the random forest and the small feature set for the prediction of V̇O_2peak_ for females. BMI: Body-Mass-Index; PAL: Physical activity level. | **Figure D.** Importance of the features by SHAP with the random forest and the small feature set for the prediction of PPO for females. BMI: Body-Mass-Index; PAL: Physical activity level. |
| 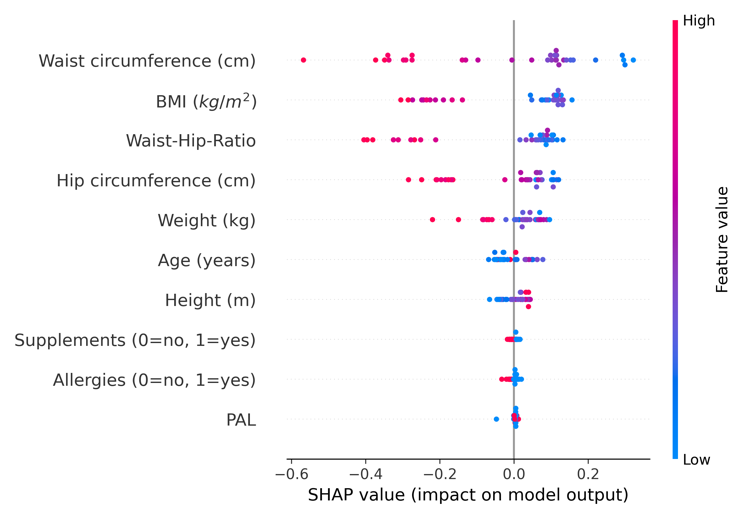 | 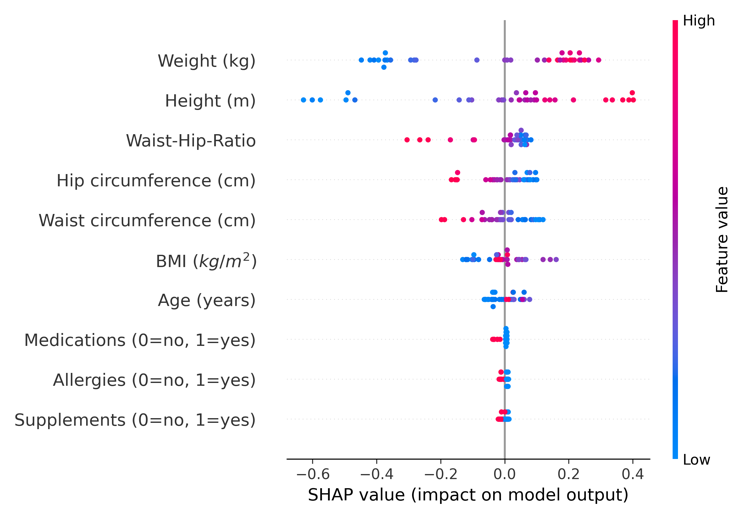 |
| **Figure E.** Importance of the features by SHAP with the random forest and the small feature set for the prediction of V̇O_2peak_ for males. BMI: Body-Mass-Index; PAL: Physical activity level. | **Figure F.** Importance of the features by SHAP with the random forest and the small feature set for the prediction of PPO for males. BMI: Body-Mass-Index; PAL: Physical activity level. |
